# New Insights into *Blastocystis* spp.: A Potential Link with Irritable Bowel Syndrome

**DOI:** 10.1371/journal.ppat.1002545

**Published:** 2012-03-15

**Authors:** Philippe Poirier, Ivan Wawrzyniak, Christian P. Vivarès, Frédéric Delbac, Hicham El Alaoui

**Affiliations:** 1 Clermont Université, Université Blaise Pascal, Laboratoire Microorganismes, Génome et Environnement, BP 10448, Clermont-Ferrand, France; 2 CNRS, UMR 6023, LMGE, Aubiere, France; 3 Clermont Université, Université d'Auvergne, JE 2526, Evolution des bactéries pathogènes et susceptibilité de l'hôte, BP 10448, Clermont-Ferrand, France; 4 CHU Clermont-Ferrand, Service Parasitologie Mycologie, Clermont-Ferrand, France; University of Wisconsin Medical School, United States of America

## What Are *Blastocystis* spp.?

*Blastocystis* spp. belong to the phylum Stramenopila, a complex and heterogeneous evolutionary assemblage of heterotrophic and photosynthetic protozoa [1]. Interestingly, this is the only stramenopile living in the lower digestive tract of humans, and it also lives in other mammals, birds, reptiles, amphibians, and insects [1]. Even though isolates were reported to be morphologically indistinguishable, an extensive genetic variation among isolates from both humans and animals has been observed. Thirteen subtypes (ST1–ST13), with the first nine being found in humans, have been identified based on genes coding for the small-subunit ribosomal RNA [2]. Preferential repartition of STs exists among animals that appear to constitute the main reservoir for environmental dissemination and human contamination [1].

Four forms of *Blastocystis* spp. (vacuolar, granular, amoeboid, and cyst) were described in stools and/or in vitro cultures [1]. Studies in animals demonstrated that the water- and environmentally resistant infective cyst undoubtedly represents the transmissible stage of this parasite [1]. *Blastocystis* spp. prevalence in humans often exceeds 5% in industrialized countries and can reach as high as 76% in developing countries [1], [3]. However, prevalence data are largely dependent on the methods used for detection, quantitative PCR being the most sensitive method, meaning that infections by *Blastocystis* spp. are likely underestimated [4].

Lately, *Blastocystis* spp. have been included in the water sanitation and health programs of the World Health Organization [5]. Increasing interest of scientific and medical communities for *Blastocystis* spp. was coupled with new data about epidemiology, pathogenicity, and, more recently, the first whole genome of a human isolate. Accumulating in vivo, in vitro, and in silico data has enabled researchers to assess the potential impact of *Blastocystis* spp. in human health.

## Are *Blastocystis* spp. Pathogens?

In vivo endoscopy and biopsy analyses in symptomatic patients indicated that *Blastocystis* spp. do not invade the colonic mucosa, but lead to disturbances on the barrier function and permeability [1], [6]. Experiments on immunocompetent BALB/c mice revealed intense inflammatory-cell infiltration in the mucosa of some specimens, but not in all mice, suggesting that some host factors could be involved [1]. Subsequently, the infectivity of human isolates obtained from both asymptomatic and symptomatic patients on rats was assessed by Hussein et al. [7]. Interestingly, the moderate and severe degrees of pathological changes were only found in rats infected by isolates from symptomatic patients, and differences in severity were observed among the different STs of *Blastocystis*, suggesting the existence of some more virulent strains.

**Figure 1:**
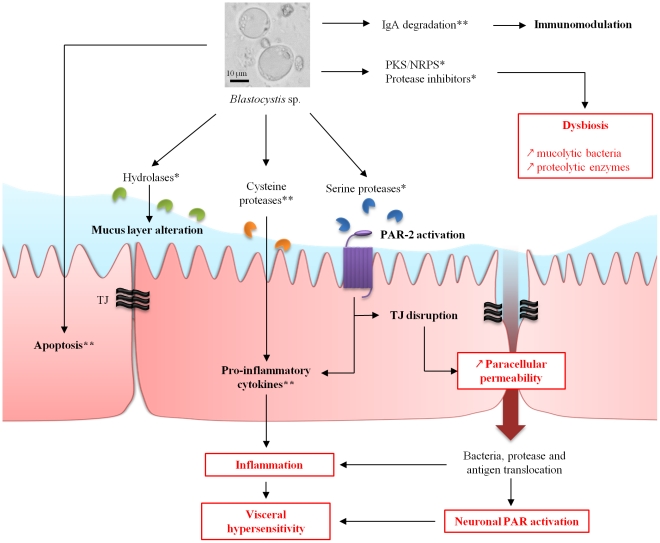
Hypothetical model of pathogeny for *Blastocystis* spp. from genomic (*) and experimental (**) data. Potential link to IBS pathophysiology mechanisms (in red). IgA, immunoglobulin A; NRPS, non-ribosomal polyketide synthase; PAR, protease-activated receptor; PKS, polyketide synthase.

To understand cellular mechanisms, in vitro experiments were performed to investigate the cytopathic effects of *Blastocystis* spp. on mammalian cell cultures (Figure 1). A first study showed in the rat epithelial cell line IEC-6 that *Blastocystis* ST4 can induce apoptosis in a contact-independent manner, increasing epithelial permeability [8]. The pro-inflammatory effect of *Blastocystis* ST1 culture filtrates was demonstrated on HT-29 and T-84 human colonic epithelial cells with production of interleukin 8 (IL-8) and granulocyte-macrophage colony stimulating factor (GM-CSF) [1]. Cysteine proteases of *Blastocystis* ST4 were shown to induce IL-8 production via an NF-κB pathway [9]. Proteases released in culture supernatants of both *Blastocystis* ST4 and ST7 were also shown to be able to cleave human-secreted immunoglobulin A (IgA) and then modulate the immune response of the host [1]. A surface-located cysteine protease was recently shown to be involved in a pro-survival role in *Blastocystis* ST7 and may activate other proteases [10]. Nevertheless, in vitro studies are limited by the lack of tools to study *Blastocystis* spp. Indeed, few strains of *Blastocystis* spp. are available in axenic cultures, and growth rates in culture are fluctuating and quite low. In addition, growth of this parasite is realized in anaerobic chambers that limit the possibility of long-term exposure in cellular models.

## Is There a Link between *Blastocystis* spp. and IBS?

Some studies have suggested an association of *Blastocystis* spp. with acute or chronic digestive disorders such as irritable bowel syndrome (IBS) [1]. IBS is a functional gastrointestinal disorder characterized by abdominal discomfort and/or pain associated with changes in bowel habits, affecting 5%–24% of people in industrialized countries with impairment on quality of life [11]. In 1997, Hussain et al. highlighted that sera from IBS patients were characterized by higher IgG antibody levels to *Blastocystis* spp. when compared to healthy populations [12]. However, the first relevant epidemiological report about a possible link between *Blastocystis* spp. and IBS was provided two years later by Giacometti et al. [13]. When comparing the prevalence of *Blastocystis* spp. in individuals with gastrointestinal symptoms and classified as affected or not by IBS, the authors found that it was significantly present in IBS patients. Other more recent studies also argued for a higher prevalence of *Blastocystis* spp. among IBS patients (Table 1) compared to healthy populations or to patients suffering from other gastrointestinal disorders [3], [13]–[17]. However, three studies failed to demonstrate an association between *Blastocystis* spp. and IBS [18]–[20]. Possible explanations could be the small IBS cohort studied or the parasitological diagnostic methods used. Two of these three studies were from Thailand and one from Mexico, whereas studies arguing for a link between *Blastocystis* spp. and IBS were from the Middle East (Table 1) and Europe. IBS is a functional disorder of multifactorial origin, and some genetic, environmental, and microbiological factors could also explain this discrepancy. Nevertheless, overall studies missed an opportunity to provide a therapeutic trial with a follow up of IBS symptoms.

**Table 1:** Summary of studies investigating the association between *Blastocystis* spp. and IBS.

Studies	Country	Diagnostic Method	IBS Patients		Control Group	
			Prevalence *n* (%)	Criteria^b^	Prevalence *n* (%)	Population
Giacometti et al. [13]	Italy	Trichrome stain	15/81 (18.5)^a^	Rome	23/307 (7.5)	GD
Yakoob et al. [17]	Pakistan	Culture	44/95 (46)^a^	Rome II	4/55 (7)	GD
Tungtrongchitr et al. [20]	Thailand	Culture	8/59 (13.6)	Rome II	3/25 (12)	Healthy
Yakoob et al. [15]	Pakistan	Culture	95/158 (60)^a^	Rome III	38/157 (24)	Healthy, GD
Surangsrirat et al. [19]	Thailand	Culture	11/66 (16.7)	Rome II	6/60 (10)	GD
Yakoob et al. [16]	Pakistan	Culture	90/171 (53)^a^	Rome III	25/159 (16)	Healthy
Dogruman-Al et al. [3]	Turkey	Lugol stain	8/21 (38)^a^	Rome III	5/43 (11.6)	Healthy
Ramirez-Miranda et al. [18]	Mexico	Flotation method	n.a./115 (15.7)	Rome III	n.a./209 (12)	GD
Jimenez-Gonzalez et al. [14]	Mexico	Flotation method	14/45 (31.1)^a^	Rome III	6/45 (13.3)	GD

a Studies concluding to an association between *Blastocystis* spp. and IBS.

b Rome criteria used to diagnose IBS.

GD, gastrointestinal disorders; n.a., not available.

Recent studies suggest that visceral pain associated with IBS could be explained by alterations of the epithelial barrier, resulting in bowel motility and sensitivity disorders (Figure 1). Indeed, in vitro studies on colonic biopsies from patients with IBS showed an increase of paracellular permeability associated with perturbations of tight junctions (TJs) [21]. On the other hand, it is now well recognized that there is a low-grade inflammation of the mucosa in IBS patients [22]. Then, protease-activated receptor type 2 (PAR-2) was proposed to be involved in both an increase of permeability and low-grade inflammation [23]. PAR-2 are activated by serine-proteases that cleave the N-terminal domain of the receptor. Then, the released peptide may act as a ligand and turn on the receptor to enhance TJ opening and trigger inflammation. Increase of paracellular permeability allows diffusion of both antigens and bacteria to sub-mucosa, participating in inflammation. PARs are also present at the surface of intestinal neurons. The activation of some members of the PAR family could contribute to abdominal pain [24]. Studies also showed that stools from IBS patients present higher proteolytic activity than healthy controls [25]. Experimental data have shown a protease activity of supernatants from axenic cultures of both *Blastocystis* ST4 and ST7 [1]. This is supported by the prediction of 22 secreted proteases from genomic data of *Blastocystis* ST7 (detailed below) [26]. These experimental and genomic data suggest a possible involvement of parasite proteases in gastrointestinal disturbances. Thus, proteases from bacteria or *Blastocystis* spp., such as metalloproteases, cysteine, or serine proteases, could play a key role in IBS genesis [24]. This perturbation could be linked to a modification of lumen microbiota in IBS patients compared to healthy patients [27]. Then, dysbiosis may take part in low-grade inflammation of the mucosa and IBS symptoms.

## How Can Genomic Data Support the *Blastocystis* spp./IBS Association?

The whole genome of a *Blastocystis* ST7 isolate has been sequenced [26]. Interestingly, candidate proteins potentially involved in the pathogenicity of *Blastocystis* spp. were identified by in silico analyses of the predicted proteome and secretome (Figure 1).

*Blastocystis* ST7 likely uses hydrolases to attack host tissues for its nutrient supply. Fucosidase, hexosaminidase, and β-galactosidase were identified in the *Blastocystis* ST7 predicted secretome. *Blastocystis* ST7 may participate in this process by degrading host glycoproteins, especially those that constitute the mucus [26]. In addition, cysteine proteases could also degrade mucins [24]. Thus, these enzymes may allow *Blastocystis* spp. to use mucus as a carbohydrate and protein source and enable them to survive within the intestinal environment by creating their own micro-environment. Interestingly, 22 proteases, including 20 cysteine proteases, one serine protease, and one aspartic protease, were predicted to be secreted [26]. The impairment of mucus may be the initial step to inflammatory and allergic disturbance caused by chronic exposure to luminal antigens. Then, proteases from *Blastocystis* spp. and/or gut bacteria can also target receptors at the intestinal cell surface. These proteolytic enzymes are known to be involved in paracellular permeability, inflammation, and hypersensitivity [24]. The *Blastocystis* ST7 serine protease could therefore have the ability to target PAR-2 (Figure 1), inducing inflammation and TJ disruption as frequently seen in IBS. Once the TJs are opened, luminal proteases can have access to submucosal ganglia, activate PARs on enteric neurons (Figure 1), and be responsible for hypersensitivity in IBS patients [24]. Penetration of luminal bacteria or antigens could participate in the establishment of a chronic low-grade inflammation in submucosa by stimulation of innate immunity.

*Blastocystis* ST7–secreted glycosyltransferases could also participate in TJ disruption in IBS. This can be illustrated by the lymphostatin (toxin) of *Citrobacter rodentium*, which possesses a glycosyltransferase activity that primarily influences localization of ZO-1 and occludin at intestinal cell TJs, compromising epithelial barrier function [24]. This is also the case for *Clostridium* spp., which exhibit toxins with glycosyltransferase activities [24]. These proteins act by inactivating Rho proteins that are known to be important in maintaining TJs.

Concerning the intestinal protease balance that regulates gut functioning, protease inhibitors released by enteric pathogens or parasites can modulate the activity of host proteases and disturb intestinal homeostasis [28]. Genes coding for protease inhibitors are also present in the *Blastocystis* ST7 genome, and some are predicted to be secreted, including cystatin, type 1-protease inhibitor, and endopeptidase inhibitor-like protein [26]. Moreover, dysbiosis was shown to occur during IBS [27]. Genomic data revealed that a polyketide synthase (PKS) and a non-ribosomal polyketide synthase (NRPS) are present in *Blastocystis* ST7. These enzymes are known to produce non-ribosomal peptides and polyketides with various biological properties, such as antibiotics or immunomodulatory molecules that could participate in dysbiosis and inflammation [29].

Finally, we can assume that *Blastocystis* spp.–secreted proteins have the potential to modulate host defenses and to facilitate nutrient acquisition and parasite colonization. Moreover, *Blastocystis* spp. would be able to alter integrity of gut epithelia and probably participate in dysbiosis.

## What Is Needed to Improve the Knowledge on *Blastocystis* spp. Biology?

A consortium including the most relevant researchers on this topic is being set up. The first aim is to develop efficient standardized tools to study *Blastocystis* spp., including the improvement of cultural methods (in particular, axenization protocols of the other STs) and development of new molecular markers for epidemiological studies. The role of STs in pathogenicity needs to be elucidated, notably by sequencing the whole genome of other STs. Comparative genomic analyses will then be useful for both identification and validation of in silico–predicted virulence factors. Animal models are also needed to go further in the understanding of the role of *Blastocystis* spp. in gut dysfunctions. Another aim is to develop some reverse genetic tools for functional characterization of genes of interest. Considering the clinical implication of *Blastocystis* spp. in IBS, none of the published studies provided complete clinical data about IBS classification and *Blastocystis* STs. Thus, well-designed multi-centric studies are required, and studies on *Blastocystis* spp. susceptibility to treatment have to be used to suggest guidelines for the eradication of this infection [30]. The role of *Blastocystis* spp. in dysbiosis and the exact interactions with bacteria also have to be highlighted. This approach seems more relevant because of the increasing interest in microbiota disturbances in the genesis of various gastrointestinal dysfunctions.
